# A major pain in the hip - Destruction of the left acetabulum and femoral head secondary to Tuberculosis: A case report and review of the literature

**DOI:** 10.1016/j.imj.2023.12.001

**Published:** 2023-12-16

**Authors:** Dominic A. Haigh, Dillan Mistry, Hamzah Z. Farooq, Katherine M.B. Ajdukiewicz

**Affiliations:** a Department of Infectious Diseases and Tropical Medicine, North Manchester General Hospital, M8 5RB Manchester, UK; b Department of Virology, Manchester University NHS Foundation Trust, M13 9WL Manchester, UK; c Faculty of Biology, Medicine and Health, University of Manchester, M13 9PL Manchester, UK

**Keywords:** Tuberculosis, Tuberculous arthritis, *Mycobacterium tuberculosis*

## Abstract

A 68-year-old-gentleman presented with left hip pain, night sweats, fatigue, and weight loss. He had previously experienced pain with white discharge until he underwent an arthroscopic washout and reduction. The left lower limb was shortened and wasted with limited hip movements. He had recently travelled to Zambia, his country of origin. Imaging demonstrated a large mass with chronic erosions of the acetabulum and femoral head. Synovial biopsy grew *Mycobacterium tuberculosis*, which was treated with rifampicin, isoniazid, pyrazinamide, and ethambutol for 2 months then 4 months of rifampicin and isoniazid. Whole genome sequencing indicated full sensitivity. Complex reconstructive surgery is scheduled, with a custom femoral head and acetabulum. This case illustrates the importance of considering tuberculosis in patients with erosive joint pathology and a multidisciplinary approach as delayed diagnosis results in high morbidity. Prompt diagnosis using newer modalities such as whole genome sequencing on synovial fluid can enable timely treatment.

## Highlights

• Tuberculosis.
• Orthopaedics.
• Multi-disciplinary team.
• Infections.

## 1 Introduction

Skeletal tuberculosis (TB) accounts for 6.7% of TB cases in England of which 2% doesn't involve the spine, and 14.4% of the extra-pulmonary cases [1]. Arthritis is an unusual presentation of TB, comprising 1%–3% of cases of TB [2]. It is commonly caused by hematogenous spread from an active or dormant pulmonary or gastrointestinal initial infection [2]. It is typically monoarthritic, with gradual onset of pain, swelling and loss of function [3]. In the USA, the proportion of TB cases that are extra-pulmonary have increased from 7.6% to 20%–40% over the last 50 years [4]. The gold standard of diagnosis is with culture of mycobacteria from biopsy or aspirate, TB PCR is also available in many centers now. Although new agents have been introduced in recent years, quadruple therapy with rifampicin, isoniazid, pyrazinamide, and ethambutol (RHZE) remains the mainstay of treatment.

We describe a case of tuberculous arthritis which resulted in the destruction of the patient's left acetabulum. We aim to explore the difficulties surrounding the diagnosis and management of an uncommon cause of infectious arthritis in a low incidence TB setting. We also aim to elaborate on how this rare case provided new insight into the management of tuberculous arthritis and the need for a multidisciplinary approach including collaboration between infectious diseases and orthopaedic surgical teams. Guidelines have been written despite a paucity of published evidence, so dialogue between specialties is crucial in forming a definitive plan of action [5].

## 2 Case presentation

A 68-year-old gentleman presented with a 2-month history of: left hip pain, fatigue, night sweats and 4 kilogram weight loss. The pain was exacerbated with movement requiring the use of a stick. The patient had returned from Zambia, his country of origin 5 days previously. He had resided in Zambia for 3 months, staying entirely within urban areas, and had not consumed bush meat. He had not knowingly been in contact with anyone with active tuberculosis, nor anyone exhibiting apparent signs of other infectious diseases including cough. He was not aware of his immunization history and there was no record from his General Practitioner. He had not taken malarial prophylaxis.

The patient fell at the age of nine years onto his left side. Since this event, he had experienced unremitting left hip pain and a white discharge from a sinus overlying the greater trochanter. It is likely that TB infection occurred as a child and seeded to the hip, this may be responsible for the weeping sinus, but this was not considered at the time. He had an arthroscopic washout and reduction 11 years prior to this presentation. The washout was initially successful with no discharge subsequently. Unfortunately, it began to occur sporadically from early adulthood.

**Fig. 1:**
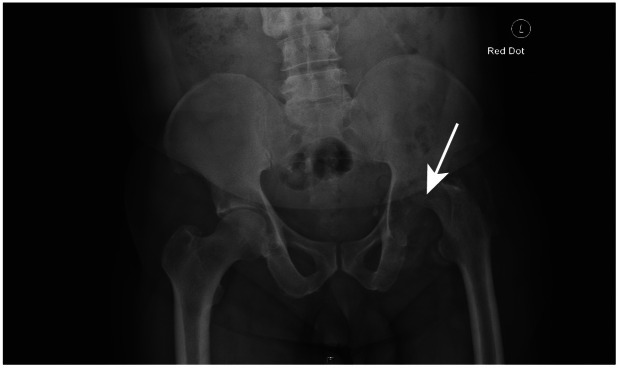
Plain film radiography of the pelvis.

The patient did not report any respiratory or gastrointestinal symptoms. His past medical history was unremarkable, apart from hypertension, for which he was treated. He did not recall being treated for febrile illness or tuberculosis in the past and denied having had any extended courses of antibiotics. The patient was unaware of family history of TB or respiratory diseases. He had a consultation with his general practitioner complaining of longstanding hip pain. Plain film radiography of the pelvis was organized as the first stage of investigation (Fig. 1). As this was grossly abnormal, he was immediately referred to be reviewed in the Emergency Department.

On admission, the patient was uncomfortable, and his mobility was limited by pain. He was pyrexial at 38℃ with his observations otherwise unremarkable and admission bloods can be found in Table 1. Clinical examination revealed generalized wasting and shortening of the left lower limb. Hip movements were globally limited, with internal rotation affected most and no neurovascular deficit. There was no cervical, axillary, or inguinal lymphadenopathy palpable. There was a scar over the left greater trochanter, but no discharge. The chest was clear to auscultation and the abdomen was soft and non-tender, with no masses palpable. There were no rashes on inspection. Initial management included taking blood cultures and malarial films. Simple analgesia was required for his left hip pain, which was titrated to effect.

**Table 1:** Admission blood test results of patient.

Blood test	Blood test results	Reference range
Hemoglobin (g/L)	117	130–180
Mean cell volume (fL)	78	80–100
White cell count (× 10^9^/L)	7.0	4.0–11.0
CRP (mg/L)	128	<10
Adjusted calcium Concentration (mmol/L)	2.67	2.10–2.60
HIV antibody	Negative	

## 3 Results

**Fig. 2:**
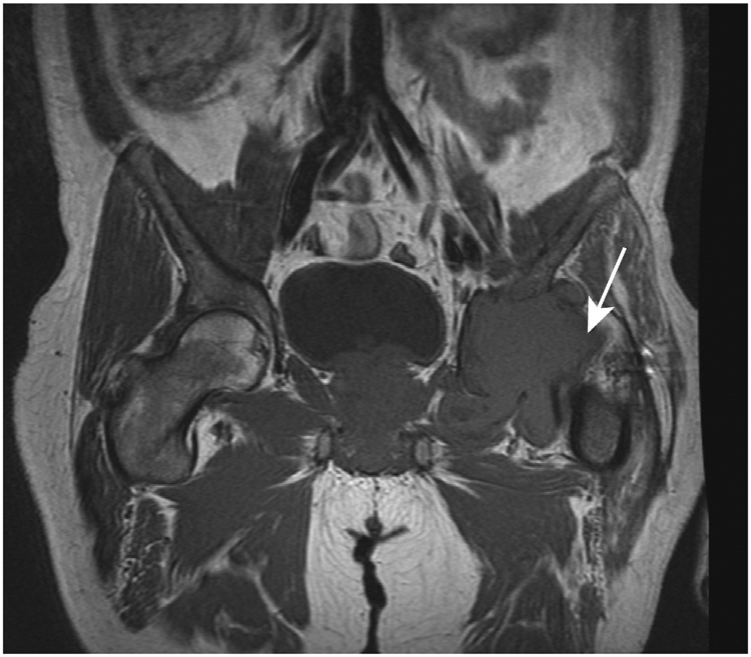
Magnetic resonance imaging of the pelvis.

Plain film radiography of the pelvis demonstrated erosions of the left acetabulum and femoral head. Plain film radiography of the chest did not demonstrate any evidence of pulmonary tuberculosis. Magnetic resonance imaging revealed a large mass with chronic erosions of the acetabulum and femoral head (Fig. 2).

The orthopaedic team reviewed the anatomical position of the destructive lesion on imaging and were concerned that it may lie in close relation to arterial vessels. As a result of this, computed tomographic angiography was requested. This showed the mass to be in close proximity to the left external iliac artery. In view of this, the orthopaedic team elected to defer operative intervention. An interferon gamma release assay performed by the referring team was positive indicating previous exposure to tuberculosis. However, this was not considered to be a useful investigation given the high suspicion of active TB infection.

Synovial biopsy was smear positive for acid-alcohol fast bacilli and both solid and liquid media grew *Mycobacterium tuberculosis* within 3 weeks. Whole genome sequencing (WGS) of the fluid demonstrated *Mycobacterium tuberculosis* (99.36% mapped to R00000039 with 2.88 million reads). The lineage was European-American, with a fully sensitive strain and no linked cases as per the WGS report and FOREST mapping on the 23rd of September 2020.

The patient was commenced on initial phase antituberculosis therapy with RIPE (with pyridoxine) for 2 months. The regime for skeletal versus pulmonary TB doesn't have any differences [6]. He was discharged home to continue treatment in the community with input from the tuberculosis specialist nurse team and follow up in the Infectious Diseases (ID) clinic; adverse reactions are common length of treatment is a balance between efficacy and reducing the rate of these. The patient completed the continuation phase of 4 further months of rifampicin and isoniazid (and pyridoxine). Following this, he was discharged from the care of the ID team. Post therapy there was a functional improvement. Routine follow up imaging has not been shown to have a beneficial effect.

Two months following the completion of anti-tuberculous therapy, complex reconstructive surgery was discussed with the patient in the orthopaedic clinic. Further CT scans with 3D reconstruction are being organized for a 3D print model of the pelvis. A custom femoral head and socket will be required for complex surgical reconstruction.

## 4 Discussion

Although tuberculosis remains a major global concern, the total number of cases of tuberculosis has fallen in England from a total of 8280 in 2011 to 4725 in 2019 [7]. Approximately 1%–3% of patients with tuberculosis have skeletal involvement [8]. Half of these cases affect the spine [6], other joints commonly affected include those of the hands, ankle, and feet [9]. The presentation of extrapulmonary tuberculosis can mimic a number of other conditions, including septic arthritis and intracranial abscesses [1].

Osteoarticular tuberculosis is classically described as having a characteristic intraoperative appearance of soft necrotic bone with underlying caseous material [10,11]. However, this is not consistently seen during arthroscopic washouts, and samples are not routinely sent for mycobacterial culture when there is a low index of suspicion.

Current clinical guidelines make no recommendation as to the optimal modality of imaging in suspected tuberculous bone or joint infection. The NICE recommendation is for 6 months of therapy in people with fully sensitive tuberculosis without central nervous system involvement [8], whereas World Health Organization and Centers for Disease Control and Prevention (CDC) guidelines recommend nine months of therapy for tuberculosis with bony or joint involvement [12,13].

Orthopaedic guidelines and expert opinion differ on the optimal timing of operative intervention with regards to anti-tuberculous chemotherapy. Traditionally, only arthrodesis and Girdlestone excision arthroplasties have been performed. Some surgeons advocate operating at the time of initiation of therapy, others following completion of the course. Total hip arthroplasties during active osteoarticular tuberculosis enable debridement of infected tissue to eradicate the disease. The concern regarding operating during the acute phase is the introduction of foreign material and the potential for biofilm formation. There is also controversy regarding the merits of one-stage versus 2-stage total hip arthroplasty [14,15].

This case demonstrates the importance of tuberculosis as a differential diagnosis in any patient with erosive hip pathology. Patients, such as in this case, are usually symptomatic for months before referral [6]. Delayed diagnosis is associated with significant morbidity including chronic pain, deformity, and disability [15]. Thus, prompt diagnosis by utilizing newer modalities such as whole genome sequencing on synovial fluid would be highly desirable in order to commence timely anti-tuberculous treatment.

Complex chronic osteoarticular infections are best managed in a collaborative manner between infectious diseases physicians, clinical microbiologists and orthopaedic surgeons. Clinical decisions are often made in individual cases in the absence of clear evidence or guidelines. In particular, the diagnosis of tuberculosis should be considered when joint infections do not respond to conventional antimicrobial regimens or have an extended course of symptoms over years.

Diagnosis was made due to multi-disciplinary working with imaging and microbiological diagnostics. As a result, the patient was appropriately treated and he functionally improved. Further research is needed to inform best practice regarding the timing and type of operative intervention and length of anti-tuberculous therapy in the management of skeletal tuberculosis.
